# *Liparisnapoensis* (Orchidaceae), a new species from Guangxi, China

**DOI:** 10.3897/phytokeys.119.32041

**Published:** 2019-03-15

**Authors:** Lin Li, Shih-Wen Chung, Bo Li, Song-Jun Zeng, Hai-Fei Yan, Shi-Jin Li

**Affiliations:** 1 Key Laboratory of Plant Resources Conservation and Sustainable Utilization, South China Botanical Garden, Chinese Academy of Sciences, Guangzhou 510650, Guangdong, China South China Botanical Garden, Chinese Academy of Sciences Guangzhou China; 2 Department of Botany, Taiwan Forestry Research Institute, Taipei, Taiwan Taiwan Forestry Research Institute Taipei Taiwan; 3 College of Agronomy, Jiangxi Agricultural University, Nanchang, 330045, Jiangxi, China Jiangxi Agricultural University Nanchang China

**Keywords:** Malaxideae, Napo County, orchid, section *Cestichis*, taxonomy

## Abstract

*Liparisnapoensis*, a new orchid species belonging to section Cestichis from Guangxi, China is described and illustrated. It occurs in the karst limestone forest. The new species is morphologically similar to *L.viridiflora* and *L.somae*, but can be readily distinguished by having narrowly oblong-falcate petals; flabellate-quadrate lip distinctly concave at base and emarginate at apex; conspicuously arcuate column with a pair of wedge-shaped wings.

## Introduction

The genus *Liparis* L.C. Richard (1817: 21), also known as false twayblade, belongs to the tribe Malaxideae of the subfamily Epidendroideae. It comprises about 320 species with cosmopolitan distribution from the tropics and subtropics to the temperate and alpine regions (Pearce and Cribb 2002, Pridgeon et al. 2005). Since its publication, various segregate genera have been proposed such as *Alatiliparis* Marg. & Szlach. (2001: 78), *Disticholiparis* Marg. & Szlach. (2004: 175), *Seidenforchis* Marg. (2006: 302) and *Platystyliparis* Marg. (2007: 35). Molecular phylogenetic studies indicated that *Liparis* in broad delimitation is polyphyletic (Cameron 2005, Li and Yan 2013). Pridgeon et al. (2005) concluded that *Liparis* sensu stricto should be restricted to a group of temperate Asian species with the type, *L.loeselii* (L.) Richard. It is as yet unclear whether the recognition of these splits provides a better taxonomy, thus we opted to maintain *Liparis* as a broad concept for the present.

As traditionally delimited, LiparissectionCestichis Thouars ex Lindley (1831: 29) is characterised by having coriaceous, non-plicate leaves. They are usually epiphytes with distinct pseudobulbs and occur mainly in tropical Asia. In China, the genus *Liparis* is represented by about 70 species (Chen et al. 2009), including eight recently described species (see Jin 2011, Wu et al. 2012, Hsu 2013, Li and Yan 2013, Su et al. 2015, Tang et al. 2015). More than half of the Chinese *Liparis* species (40, about 57%) belong to section Cestichis.

The limestone karst area is part of a global biodiversity hotspot. Floristic investigations of limestone areas in southwestern Guangxi, China from April 2012 to July 2015, have yielded the discovery of an interesting Liparisspecies of section Cestichis. Morphologically, this species is superficially similar in appearance to *L.viridiflora* (Blume) Lindley (1831: 31) in vegetative habit, but differs significantly from the latter in the floral morphology. On the other hand, it bears yellowish or whitish flowers somewhat resembling those of *L.somae*Hayata (1914: 33), a rare species endemic to Taiwan, but several critical details differ. Over the past three years, the living and cultivated specimens were monitored in the field and in the nursery of South China Botanical Garden (SCBG), Chinese Academy of Sciences (CAS). Careful examinations of diagnostic morphological features of similar taxa and literature surveys (Seidenfaden 1976, Comber 1990, Yang 2006, Averyanov 2013, 2015, Tang et al. 2015) indicated that it represents a species new to science and accordingly described herein.

## Materials and method

A total of 450 herbarium specimens of Liparis species in the section Cestichis were examined from herbaria BM, E, HN, IBK, IBSC, K, PE and US (acronyms according to Thiers 2018). The taxonomic status of *Liparisnapoensis* and its close allies were examined by checking the type specimens in these herbaria and online digital image repositories and databases available on JSTOR Global Plants website (http://plants.jstor.org). Relevant literature, including protologue was consulted. Morphological descriptions and measurements of the putative new species were undertaken based on three living specimens in cultivation (South China Botanical Garden). The specimens were observed and photographed under a stereomicroscope (Olympus MD-90). The conservation status of the new species was evaluated following the guidelines in IUCN (2017).

## Taxonomic description

### Liparis napoensis

Taxon classificationPlantaeAsparagalesOrchidaceae

L.Li, H.F.Yan & S.J.Li sp. nov.

urn:lsid:ipni.org:names:60478421-2

Figures 1 , 2

#### Type.

CHINA. Guangxi Zhuang Autonomous Region, Baise City, Napo County, Yongning Village, Jinlongyan Cave, lithophytic on moss in monsoon evergreen broad-leaved forest, alt. 830 m, 31 May 2012, L. Li 1001, (holotype, IBSC!).

#### Diagnosis.

*Liparisnapoensis* differs from its closest allies: *L.viridiflora* and *L.somae* in the clustered ovoid-cylindrical or narrowly pyriform pseudobulbs, narrowly oblong-falcate petals; a flabellate-quadrate lip with a distinctly concave base, an emarginate apex and erose margins; a conspicuously arcuate column with a pair of wedge-shaped wings.

#### Description.

Lithophytic herbs. Pseudobulbs clustered, ovoid-cylindrical or narrowly pyriform, 5–10 cm × 5–8 mm, attenuate toward apex, base covered with 3–4 fibrous remnant sheaths. Leaves 2, terminal, blade oblanceolate or oblong-spatulate, thin coriaceous, base contracted into a very short petiole less than 1 cm or subsessile, apex acute and minutely apiculate, 7–12× 1.5–2 cm. Inflorescence terminal, often recurved, 15–20 cm, densely racemose, pedunculate; rachis 10–15 cm with densely arranged numerous flowers; floral bracts linear-lanceolate, 3.5–4 mm, membranous, greenish-white. Flowers resupinate, spreading, 4–4.5 mm across, white, tinged with pale yellow in the centre; pedicel and ovary 3.5–4 mm, pale yellow or greenish-yellow. Dorsal sepal ovate-oblong, margins often revolute, apex obtuse, 2.5–3 × 0.8–1 mm, translucent white, becoming yellowish towards the base. Lateral sepals obliquely ovate-elliptic, margins often revolute, 2.5–3 × 0.8–1 mm, translucent white, becoming yellowish towards the base. Petals narrowly oblong-falcate, margins revolute, 2–2.5 × 0.3–0.5 mm, translucent white, becoming yellowish towards the base. Lip nearly flabellate-quadrate, 2–2.5 × 1.5–2 mm, base shallowly concave and distinctly recurved from the middle, indistinctly divided into hypochile and epichile; hypochile ecallose, adaxially slightly thickened and fleshy towards the margins on each side; epichile emarginate, apical margins inconspicuously erose, translucent white, furnished with pale yellow at base. Column terete, conspicuously incurved or arcuate above the middle, apex with 2 short, wedge-shaped wings, with the base slightly dilated, 1.0–1.5 mm long, greenish-white, tinged with pale yellow at base. Stigma concave, subelliptic. Rostellum approximately truncate, apex obtuse, yellowish. Anther terminal, 2-celled, persistent, compressed ovoid, ca. 0.5 mm long. Pollinia 4, hard, waxy, yellow, ca. 0.3 mm. Capsule obovoid-ellipsoid, ridged, 4–6 × 2–3 mm; fruiting pedicel 3–5 mm.

**Figure 1.**
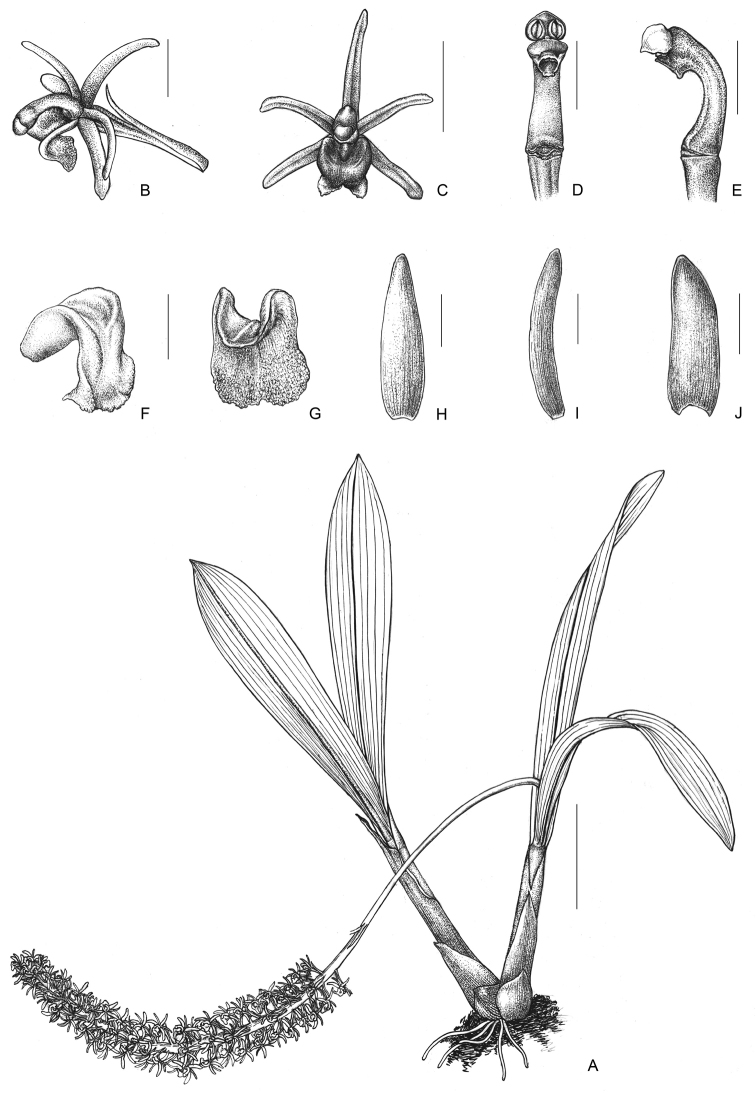
*Liparisnapoensis***A** Habit in bloom **B** Flower, lateral view **C** Flower, front view **D** Column, ventral view **E** Column, lateral view **F, G** Lip **H** Dorsal sepal **I** Petal **J** Lateral sepal. Line drawing by Yun-Xiao Liu. Scale bars: 2.5 cm (**A**), 2 mm (**B, C**), 1 mm (**D–I**).

**Figure 2.**
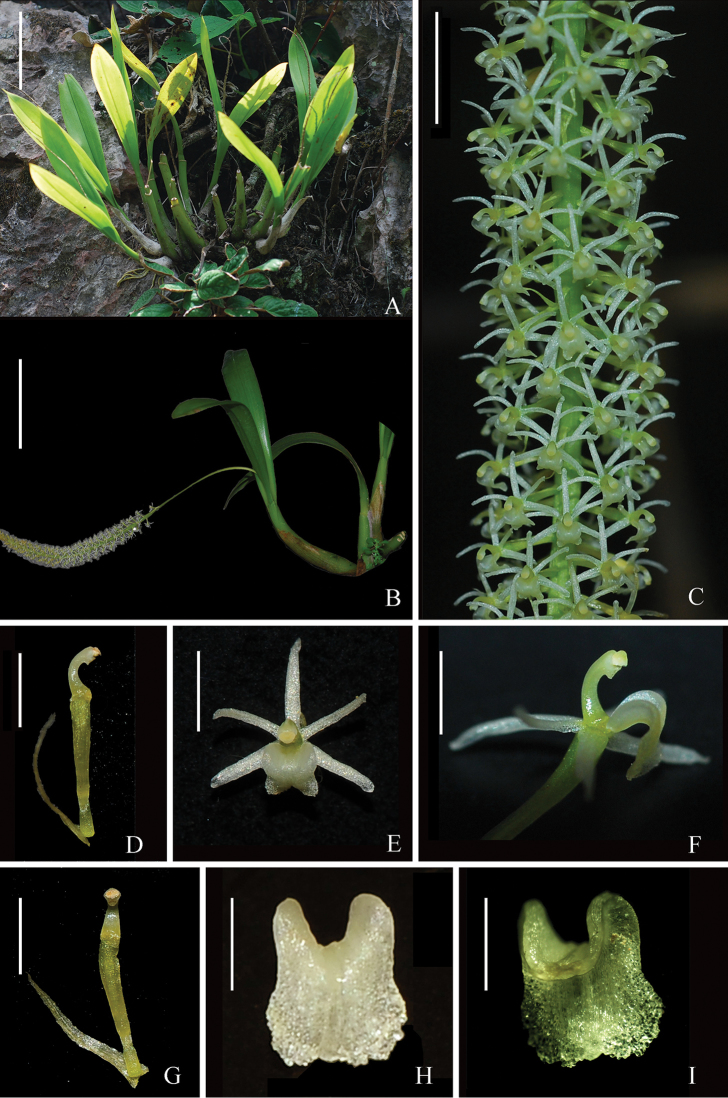
*Liparisnapoensis***A** Habitat **B** A plant in bloom **C** Inflorescence **D** Column, lateral view, showing bract **E** Flower, frontal view **F** Flower, lateral view **G** Column, ventral view, showing bract **H, I **Lip. Scale bars: 5 cm (**A–C**), 2 mm (**D–G**), 1 mm (**H, I**).

#### Distribution and habitat.

Endemic to Guangxi in China. Lithophytes in rocks crevices where soil or organic matter has accumulated at elevations from ca. 650 m to 900 m in karst limestone forest.

#### Etymology.

The epithet “napoensis” is derived from the type locality: Napo County, Guangxi, located at China’s southwest border, where the species was discovered.

#### Phenology.

Flowering and fruiting in January–February.

#### Conservation status.

Based on careful field investigations in the past years, this species is rare and only known from the type locality. Plants grow in sparsely scattered groups and the known population of two colonies consists of only a few dozen individuals (density less than one plant per 20 m^2^). In addition, the location is not in a protected area and accessible to casual hikers. According to the guidelines for using the IUCN Red List Categories and Criteria (IUCN 2017), the species is categorised as Critically Endangered [CR B2ab(iii)] due to its rarity and the threat of disturbance.

#### Taxonomic notes.

The new species in its general appearance has some superficial similarity with *Liparisviridiflora*, but the latter differs in longer petioles, narrowly linear petals, ovate-oblong lip with a mucronate tipped apex and a column with rounded wings. It also superficially resembles *L.somae*, but is characterised by having ovoid-cylindrical or narrowly pyriform pseudobulbs, a flabellate-quadrate lip with a distinctly concave base and an emarginate apex. A detailed morphological comparison between *L. napoensis* and its closely related taxa *L.viridiflora* and *L.somae* is presented in Table 1.

**Table 1.** Comparison of the diagnostic characters of *L.napoensis* and its allies.

Characters	* L. napoensis *	* L. somae *	* L. viridiflora *
Growth habit	lithophytes	epiphytes	epiphytes or lithophytes
Pseudobulbs	ovoid-cylindrical or narrowly pyriform	ovoid or clavate	elongate-cylindrical
Leaves	oblanceolate or oblong-spatulate, apex acute and minutely apiculate	oblanceolate or spatulate, apex acute	linear-oblanceolate, apex acuminate and apiculate
Petiole	less than 1 cm or subsessile	2–3 cm	1–4 cm
Dorsal sepal	ovate-oblong	lanceolate	elliptic-oblong
Lateral sepals	obliquely ovate-elliptic	obliquely ovate	ovate-elliptic
Petals	narrowly oblong-falcate	linear	narrowly linear
Lip	flabellate-quadrate, base concave, slightly thickened on each side, apex emarginate, apical margin inconspicuously erose	ovate, base slightly contracted, apex acute, apical margin slightly crisped-denticulate	ovate-oblong, base contracted, apex subacute or mucronate, apical margin slightly undulate
Column	conspicuously arcuate, apex with 2 wedge-shaped wings	slightly arcuate, apex with 2 obtusely dentate wings	slightly arcuate, apex with rounded wings

## Supplementary Material

XML Treatment for Liparis napoensis
